# ANGPTL4 mediates the protective role of PPAR*γ* activators in the pathogenesis of preeclampsia

**DOI:** 10.1038/cddis.2017.419

**Published:** 2017-09-21

**Authors:** Lei Liu, Xu Zhuang, Meng Jiang, Fei Guan, Qin Fu, Jianhua Lin

**Affiliations:** 1 Department of Obstetrics and Gynecology, Ren Ji Hospital, School of Medicine, Shanghai Jiao Tong University, Shanghai, China; 2 Shanghai Key Laboratory of Gynecologic Oncology, Ren Ji Hospital, School of Medicine, Shanghai Jiao Tong University, Shanghai, China

## Abstract

Peroxisome proliferator-activated receptor *γ* (PPAR*γ*) has been shown to be a therapeutic target for preeclampsia (PE). Angiopoietin-like protein 4 (ANGPTL4) is a multifunctional secretory protein involved in regulating lipid metabolism and angiogenesis in various tissues. However, the expression of PPAR*γ* and ANGPTL4 and their interaction in PE remain elusive. Here we showed that PPAR*γ* agonist rosiglitazone upregulated the expression and secretion of ANGPTL4 in a dose-dependent manner in HTR8/SVneo cells, human umbilical vein endothelial cells (HUVECs) and placental explants. More importantly, we confirmed that the PPAR*γ*/retinoid X receptor *α* heterodimer specifically binds to the ANGPTL4 promoter region and enhances its transcriptional activity. In addition, the levels of ANGPTL4 and PPAR*γ* activators in the serum and their expression in placental tissues were significantly reduced in preeclamptic patients compared with normal pregnant subjects. Furthermore, functional studies demonstrated that ANGPTL4 mediates the facilitative effects of the PPAR*γ* agonist on the survival, proliferation, migration and invasion of HTR8/SVneo cells, placental explants outgrowth and angiogenesis in HUVECs. Taken together, our results suggest that ANGPTL4 is a potential target gene for PPAR*γ* and mediates the protective role of PPAR*γ* activators in the pathogenesis of PE.

Preeclampsia (PE) is a pregnancy-specific disorder in humans that serves as a predominant contributor to maternal mortality and affects, approximately 2–8% of pregnancies around the world.^1^ Although the cause and pathophysiology of PE remain largely unclear, it is generally accepted that the placenta is of great importance in the pathogenesis of PE because removal of the placenta can eradicate clinical symptoms in the patients with PE.^2^ At present, a growing body of evidence indicates that abnormally shallow placentation in early pregnancy is mainly responsible for the onset of PE.^3^ Excessive apoptosis of trophoblast cells, poor invasion of the uterine wall by trophoblasts and impaired remodelling of spiral arteries at the maternal–foetal interface are major abnormal placentation events that are closely related to PE. A wide range of growth factors and hormones are also believed to be involved in the intricate regulation of these events.

Peroxisome proliferator-activated receptors, including PPAR*α*, PPAR*β/δ* and PPAR*γ*, are ligand-activated transcription factors that regulate a number of genes associated with cell differentiation and proliferation.^4^ PPAR*γ* has an important role in the differentiation of complicated trophoblast lineages and normal vascular function.^5, 6, 7^ In addition, recent studies have shown that serum concentrations of endogenous activators of PPAR*γ* are dramatically decreased in severe PE patients compared with healthy pregnant women,^8^ and PPAR*γ* may serve as a novel therapeutic target for PE.^9^ Nevertheless, the molecular mechanisms of the protective roles of PPAR*γ* in PE remain largely unknown.

Angiopoietin-like protein 4 (ANGPTL4), a secretory glycoprotein, is a member of the angiopoietin family.^10^ Previous studies have suggested that ANGPTL4 is a multifunctional factor involved in the regulation of lipid metabolism, wound healing and angiogenesis.^11, 12^ Furthermore, it has been reported that activation of PPAR*γ* induces the expression and secretion of ANGPTL4.^13, 14^ However, the expression and secretion of ANGPTL4 in PE has not been investigated. In addition, it remains unknown whether a regulatory interaction between ANGPTL4 and PPAR*γ* exists in PE.

Here, we investigated the effects of the PPAR*γ* agonist rosiglitazone on the expression and secretion of ANGPTL4 and the molecular mechanisms underlying these effects in HTR8/SVneo cells, human umbilical vein endothelial cells (HUVECs) and placental explants. Moreover, we explored the expression of ANGPTL4 and PPAR*γ* in placental tissue and serum as well as their potential correlation in preeclamptic patients and healthy subjects. To further identify the latent roles of ANGPTL4 and PPAR*γ* in PE, a variety of functional studies were performed using cell lines and placental explant models.

## Results

### PPAR*γ* is indispensable for the rosiglitazone-induced expression and secretion of ANGPTL4

**Figure 1:**
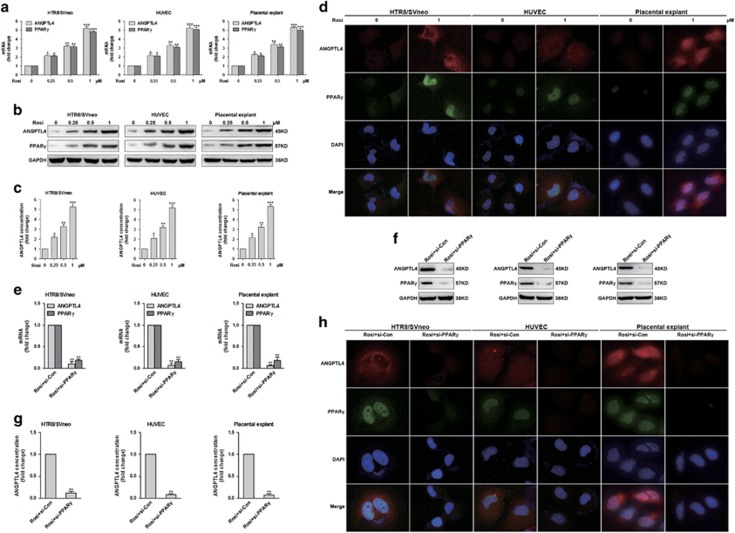
Rosiglitazone stimulates the expression and secretion of ANGPTL4 via PPAR*γ*. (**a**–**c**) HTR8/SVneo cells, HUVECs and placental explants were stimulated with Rosi (rosiglitazone) (0, 0.25, 0.5 and 1 *μ*M) for 18 h. The expression of PPAR*γ* and ANGPTL4 and the secretion of ANGPTL4 were determined by quantitative real-time PCR (qRT-PCR), western blot and enzyme-linked immunosorbent assay (ELISA). The data are shown as the means±S.E.M. *P<0.05, **P<0.01 and ***P<0.001 compared with control. (**d**) HUVECs, HTR8/SVneo cells and placental explants were stimulated with different concentrations (0 and 1 *μ*M) of rosiglitazone for 18 h, and then stained to detect the expression of PPAR*γ* and ANGPTL4 by immunofluorescence. (**e**–**h**) HTR8/SVneo cells, HUVECs and placental explants were transfected with control siRNA (si-Con) or PPAR*γ* siRNA (si-PPAR*γ*), and then treated with 1 *μ*M rosiglitazone for 18 h. The expression of PPAR*γ* and ANGPTL4 and the secretion of ANGPTL4 were evaluated by qRT-PCR, western blot analysis, ELISA and immunofluorescence. The data are shown as the means±S.E.M. **P<0.01 relative to corresponding control

To examine the role of the PPAR*γ* agonist rosiglitazone in the expression and secretion of ANGPTL4, HTR8/SVneo cells, HUVECs and placental explants were stimulated with different concentrations (0, 0.25, 0.5 and 1 *μ*M) of Rosi (rosiglitazone). As shown in Figures 1a and c and Supplementary Figure 1a, rosiglitazone-induced ANGPTL4 mRNA and protein expression and its secretion in a concentration-dependent manner. Rosiglitazone also induced the mRNA and protein expression of PPAR*γ* in a similar manner (Figures 1a–c). The rosiglitazone-induced expression of ANGPTL4 and PPAR*γ* was also demonstrated by immunofluorescence staining (Figure 1d).

To further determine the role of PPAR*γ* in the rosiglitazone-induced expression and secretion of ANGPTL4, HTR8/SVneo cells, HUVECs and placental explants were transfected with control siRNA (si-Con) or PPAR*γ* siRNA (si-PPAR*γ*) and treated with 1 *μ*M rosiglitazone. The results showed that silencing of PPAR*γ* expression inhibited the effects of rosiglitazone on ANGPTL4 protein and mRNA expression as well as its secretion (Figures 1e–g and Supplementary Figure 1b). The same results were confirmed in the cell lines and explants through immunofluorescence staining (Figure 1h and Supplementary Figure 1c). These data suggest that PPAR*γ* is indispensable for the rosiglitazone-induced expression and secretion of ANGPTL4.

### ANGPTL4 is a direct transcriptional target of PPAR*γ*

**Figure 2:**
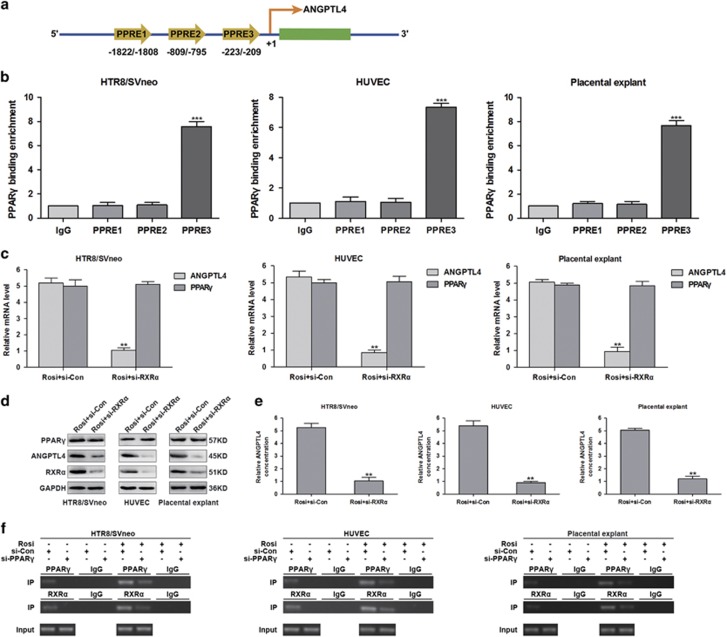
The PPAR*γ* agonist induces ANGPTL4 expression and secretion by promoting the binding of the PPAR*γ*/RXR*α* heterodimer to its promoter. (**a**) Schematic representation of the ANGPTL4 gene promoter showing the location of the potential peroxisome proliferator-responsive elements (PPREs). (**b**) Chromatin immunoprecipitation (ChIP) assays were performed in HTR8/SVneo cells, HUVECs and placental explants using antibodies against PPAR*γ* or IgG. Purified DNA was detected by qRT-PCR using primer sets specific to PPRE1-PPRE3 of ANGPTL4. The data are shown as the means±S.E.M. ***P<0.001 relative to IgG. (**c**–**e**) HTR8/SVneo cells, HUVECs and placental explants were transfected with control siRNA (si-Con) or RXR*α* siRNA (si-RXR*α*) and then stimulated with 1 *μ*M rosiglitazone for 18 h. The mRNA and protein expression of PPAR*γ* and ANGPTL4, the protein expression of RXR*α* and the secretion of ANGPTL4 were detected via qRT-PCR, western blot analysis and ELISA. The data are shown as the means±S.E.M. **P<0.01 *versus* control. (**f**) HUVECs, HTR8/SVneo cells and placental explants were transfected with si-Con or si-PPAR*γ* and then treated with or without 1 *μ*M rosiglitazone for 18 h. ChIP assays were accomplished with specific antibodies against PPAR*γ* and RXR*α*

Activated PPAR*γ* regulates gene expression via heterodimerizing with retinoid X receptors (RXRs) and binding to the peroxisome proliferator-responsive element (PPRE) of target genes.^15^ Given that PPAR*γ* is implicated in the regulation of ANGPTL4 expression and secretion, we speculated that a PPRE likely exists in the promoter region of ANGPTL4 for its transactivation. To test this hypothesis, we analysed the human ANGPTL4 5'-flanking region and identified three putative PPREs, namely PPRE1 (−1822/−1808), PPRE2 (−809/−795) and PPRE3 (−233/−209), located upstream of the ANGPTL4 transcription start site (Figure 2a). Next, to determine whether PPAR*γ* binds to these regions, a chromatin immunoprecipitation (ChIP) assay was performed in HTR8/SVneo cells, HUVECs and placental explants. The results indicated that PPAR*γ* binds to PPRE3, located at −233/−209 (Figure 2b).

The PPAR*γ*/retinoid X receptor *α* (PPAR*γ*/RXR*α*) heterodimer is required for placental development.^16^ To investigate whether RXR*α* is involved in the rosiglitazone-induced expression and secretion of ANGPTL4, HTR8/SVneo cells, HUVECs and placental explants were transfected with control siRNA (si-Con) or RXR*α* siRNA (si-RXR*α*) and treated with rosiglitazone. As shown in Figures 2c–e, silenced RXR*α* expression abolished the effects of rosiglitazone on ANGPTL4 protein and mRNA expression, as well as its secretion. Surprisingly, the rosiglitazone-induced PPAR*γ* protein and mRNA expression were not affected by treatment with si-RXR*α* (Figures 2c and d). These results demonstrate that RXR*α* is a prerequisite for rosiglitazone-induced ANGPTL4 expression. Subsequently, to further determine whether RXR*α* also binds to the PPRE3 in ANGPTL4 promoter region, ChIP assays were carried out in HTR8/SVneo cells, HUVECs and placental explants. The data showed that both PPAR*γ* and RXR*α* bound to PPRE3 in the ANGPTL4 promoter (Figure 2f). Together, these results indicate that rosiglitazone stimulates ANGPTL4 expression via regulating the binding of the PPAR*γ*/RXR*α* heterodimer to its promoter.

### The expression and circulating activators of PPAR*γ* and the expression and secretion of ANGPTL4 are decreased in PE

**Figure 3:**
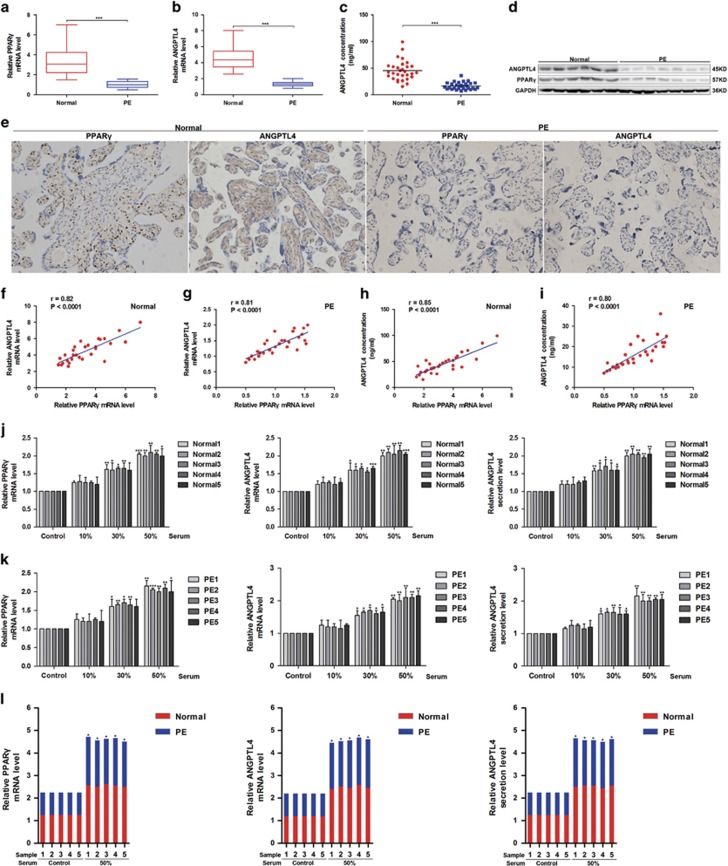
The expression and secretion of ANGPTL4 are decreased and positively correlated with the expression of PPAR*γ* in PE. (**a**, **b**) The mRNA levels of PPAR*γ* and ANGPTL4 in placental tissues were detected in PE (*n*=30) and normal controls (*n*=30) by qRT-PCR. ***P<0.001 against normal controls. (**c**) The secretion of ANGPTL4 in the serum was measured among PE (*n*=30) and normal controls (*n*=30) by ELISA. ***P<0.001 compared with normal controls. (**d**) The expression of ANGPTL4 and PPAR*γ* in placental tissues was determined by western blot analysis in seven PE subjects and six normal controls randomly selected from 60 placental tissues. (**e**) Immunohistochemistry (IHC) was performed to analyse the expression of PPAR*γ* and ANGPTL4 in PE subjects (*n*=30) and normal controls (*n*=30). (**f**–**i)** The relationship between the mRNA expression of PPAR*γ* and the mRNA and secretion of ANGPTL4 was analysed in PE subjects (*n*=30) and normal controls (*n*=30). (**j**, **k**) Placental explants were stimulated with serum from PE subjects (*n*=5) and normal controls (*n*=5) randomly selected from 60 blood samples. PPAR*γ* and the mRNA and secretion of ANGPTL4 were assessed by qRT-PCR and ELISA. The data are shown as the means±S.E.M. *P<0.05, **P<0.01 and ***P<0.001 compared with corresponding control. (**l**) The combined data from (**j**, **k**) was analysed. The data are shown as the means. *P<0.05 compared with PE

As shown in Supplementary Table 1, there were no obvious differences in body mass index, age, gestational age and infant birth weight between normal pregnant (*n*=30) and preeclamptic women (*n*=30) enroled in this study. Next, to assess the physiological significance of PPAR*γ* and ANGPTL4 in placental development, we detected their mRNA and protein expression among placental tissues by western blot, quantitative real-time PCR (qRT-PCR) and immunohistochemistry (IHC) analyses. The results showed that the mRNA and protein expression levels of PPAR*γ* and ANGPTL4 were significantly decreased in PE samples compared with normal controls (Figures 3a). The secretion of ANGPTL4 in the serum was also decreased in PE subjects compared with normal controls (16.2±1.2 *versus* 44.9±3.4 ng/ml, ***P<0.001) (Figure 3c).

Because the PPAR*γ* agonist rosiglitazone induces the mRNA and protein expression of PPAR*γ* in a concentration-dependent way in placental explants, we speculated that circulating activators of PPAR*γ* were decreased in PE subjects compared with normal pregnant women. To prove this hypothesis, placental explants were treated with serum from normal controls (*n*=5) and PE subjects (*n*=5). The mRNA expression of PPAR*γ* and ANGPTL4 as well as the secretion of ANGPTL4 were upregulated by serum from both PE and control subjects in a concentration-dependent manner (Figures 3j and k) and increased in normal pregnant women compared with PE (Figure 3l and Supplementary Figure 2). In addition, PPAR*γ* mRNA was significantly reduced in preeclamptic placentae compared with normal placentae (Figure 3a). Based on the above results, our hypothesis that circulating activators of PPAR*γ* in PE are decreased may be correct.

Next, correlation analysis revealed a positive relationship between the mRNA level of PPAR*γ* and the mRNA level and secretion of ANGPTL4 in PE (*n*=30) and normal pregnant women (*n*=30) (Figures 3f–i), suggesting a functional interaction between PPAR*γ* and ANGPTL4 *in vivo*. Given that serum from PE and control subjects stimulated PPAR*γ* mRNA expression in a concentration-dependent manner, the mRNA level of PPAR*γ* in placental tissues should be an indicator of circulating activators of PPAR*γ* in serum. Together with the results shown in Figures 3f–i, we hypothesised that circulating PPAR*γ* activators positively correlate with the secretion of ANGPTL4 in normal pregnant and PE women. Taken together, the expression and circulating activators of PPAR*γ* and the expression and secretion of ANGPTL4 are decreased in PE, and the reduced expression and secretion levels of ANGPTL4 may result from a decrease in circulating activators of PPAR*γ* in serum.

### ANGPTL4 mediates rosiglitazone-induced trophoblast cell survival and proliferation

**Figure 4:**
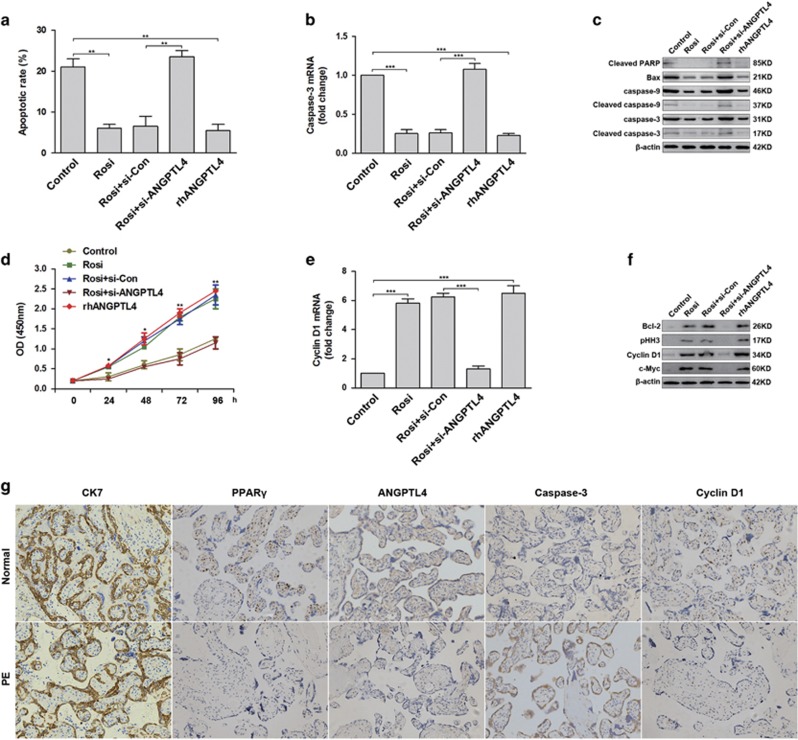
ANGPTL4 is essential for rosiglitazone-induced trophoblast cell survival and proliferation. (**a**–**c**) HTR8/SVneo cells transfected with control siRNA (si-Con) or ANGPTL4 siRNA (si-ANGPTL4) were stimulated with 1 *μ*M rosiglitazone in the presence of 150 *μ*M hydrogen peroxide. Control cells were treated with 1 *μ*M rosiglitazone or 100 nM recombinant human ANGPTL4 (rhANGPTL4) in the presence of 150 *μ*M hydrogen peroxide. A TUNEL assay was performed to evaluate the rate of cellular apoptosis. Caspase-3 expression was detected by qRT-PCR, and the expression of caspase-9, cleaved caspase-9, caspase-3, cleaved caspase-3, cleaved PARP and Bax was measured via western blot analysis. The data are shown as the means±S.E.M. **P<0.01, ***P<0.001 compared with control or si-Con. (**d**–**f**) HTR8/SVneo cells transfected with si-Con or si-ANGPTL4 were stimulated with 1 *μ*M rosiglitazone. Control cells were treated with 1 *μ*M rosiglitazone or 100 nM rhANGPTL4. A CCK-8 assay was performed to examine cell proliferation. Cyclin D1 mRNA was assessed by qRT-PCR, and the expression of Bcl-2, pHH3, Cyclin D1 and c-Myc was determined by western blot analysis. The data are shown as the means±S.E.M. *P<0.05, **P<0.01 and ***P<0.001 against control or si-Con. (**g**) The expression of CK7, PPAR*γ*, ANGPTL4, Cyclin D1 and caspase-3 in placental tissues was further analysed by IHC in PE subjects (*n*=30) and normal controls (*n*=30). Representative images were captured at × 200 magnification

Excessive apoptosis of trophoblast cell is a major abnormal placentation event involved in PE.^3^ Oxidative stress is responsible for increased trophoblast cell apoptosis.^17^ Therefore, we determined the effects of ANGPTL4 and PPAR*γ* on trophoblast cell survival. HTR8/SVneo cells transfected with control siRNA (si-Con) or ANGPTL4 siRNA (si-ANGPTL4) were treated with 150 *μ*M hydrogen peroxide and 1 *μ*M rosiglitazone for 48 hours. Controls were stimulated with 150 *μ*M hydrogen peroxide and 100 nM recombinant human ANGPTL4 (rhANGPTL4). Subsequently, terminal deoxynucleotidyl transferase-mediated deoxyuridine triphosphate nick end labelling (TUNEL) assays were performed to determine the rate of cellular apoptosis. Rosiglitazone and rhANGPTL4 dramatically inhibited trophoblast cell apoptosis induced by hydrogen peroxide, and the deletion of ANGPTL4 abolished the anti-apoptotic effects of rosiglitazone (Figure 4a and Supplementary Figure 3). At the same time, the expression of molecular markers associated with apoptosis, such as Bax, cleaved PARP, caspase-9, cleaved caspase-9, caspase-3 and cleaved caspase-3, was also reduced in cells treated with rosiglitazone and rhANGPTL4 compared with the control (Figures 4b and c). Furthermore, the expression of CK7 (a marker of trophoblast), PPAR*γ*, ANGPTL4 and caspase-3 was assessed through IHC in preeclamptic placentae and normal controls. The results indicated that caspase-3 expression was increased in preeclamptic placentae with lower PPAR*γ* and ANGPTL4 expression compared with normal placentae (Figure 4g).

Next, the roles of ANGPTL4 and PPAR*γ* in trophoblast cell proliferation were assessed using a cell proliferation assay. Similarly, rosiglitazone and rhANGPTL4 significantly promoted trophoblast cell proliferation compared with controls, and ablation of ANGPTL4 blocked the effects of rosiglitazone (Figure 4d). In addition, the expression of cell proliferation-related genes, such as Cyclin D1, Bcl-2, c-Myc and pHH3, was clearly upregulated in cells treated with rosiglitazone and rhANGPTL4 compared with controls (Figures 4e and f). Consistent with these results, decreased expression of PPAR*γ*, ANGPTL4 and cyclin D1 was observed in PE placentae compared with controls by IHC (Figure 4g). In conclusion, these data indicate that ANGPTL4 is essential for the PPAR*γ* agonist-induced survival and proliferation of trophoblasts.

### ANGPTL4 is important for the migration and invasion of trophoblast cell and placental explant outgrowth induced by the PPAR*γ* agonist

**Figure 5:**
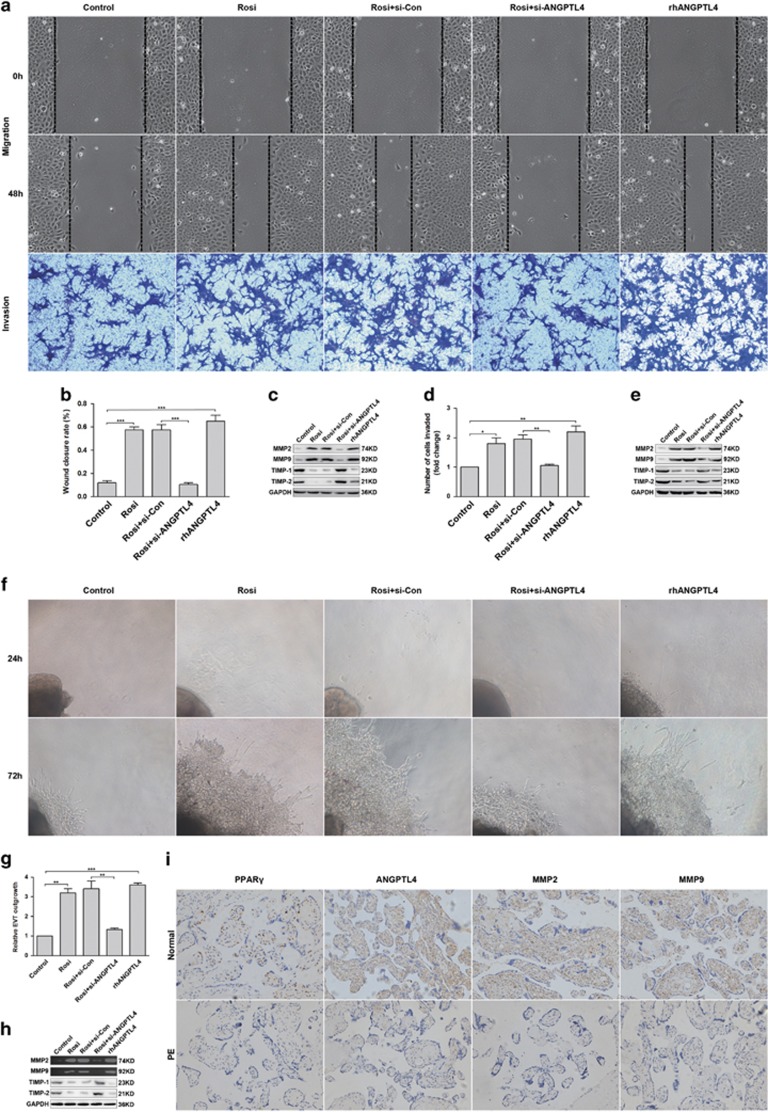
ANGPTL4 meditates PPAR*γ* agonist-induced migration and invasion of trophoblast cells and outgrowth of placental explants. (**a**–**e**) HTR8/SVneo cells transfected with si-Con or si-ANGPTL4 were stimulated with 1 *μ*M rosiglitazone. Control cells were treated with 1 *μ*M rosiglitazone or 100 nM rhANGPTL4. Transwell invasion and wound-healing assays were performed to assess the invasive and migratory abilities of these cells. The expression of MMP-2, MMP-9, TIMP-1 and TIMP-2 was tested via western blot analysis. The data are shown as the means±S.E.M. *P<0.05, **P<0.01 and ***P<0.001 *versus* control or si-Con. (**f**–**h**) Placental explants transfected with si-Con or si-ANGPTL4 were treated with 1 *μ*M rosiglitazone. Control explants were stimulated with 1 *μ*M rosiglitazone or 100 nM rhANGPTL4. Placental explant outgrowth was measured to further assess extravillous cytotrophoblast (EVT) migration and invasion. Representative images of placental explants are shown. The expression of MMP-9 and MMP-2 was measured via gelatin zymography, and the expression of TIMP-1 and TIMP-2 was determined by western blot analysis. The data are shown as the means±S.E.M. **P<0.01, ***P<0.001 compared with control or si-Con. (**i**) The expression of PPAR*γ*, ANGPTL4, MMP-2 and MMP-9 in placental tissues was determined by IHC in PE subjects (*n*=30) and normal controls (*n*=30). Representative images were captured at × 200 magnification

To elucidate the roles of ANGPTL4 and PPAR*γ* in trophoblast cells migration and invasion, transwell invasion and wound-healing assays were executed in HTR8/SVneo cells transfected with si-ANGPTL4 or si-Con and control cells. Rosiglitazone and rhANGPTL4 exhibited similar roles in the migration and invasion of trophoblast cells compared with control cells, and silenced ANGPTL4 abolished the effects of rosiglitazone (Figures 5a). To determine the mechanisms by which rosiglitazone and ANGPTL4 act synergistically to promote trophoblast cells migration and invasion, we investigated their roles in matrix metalloproteinase-2 (MMP-2) and matrix metalloproteinase-9 (MMP-9) expression. Compared with control cells, rosiglitazone and rhANGPTL4 significantly increased the expression of MMP-2 and MMP-9, accompanying with decreased tissue inhibitor of metalloproteinase-1 (TIMP-1) and tissue inhibitor of metalloproteinase-2 (TIMP-2) expression (the tissue inhibitors of MMP-9 and MMP-2) (Figures 5c and e).

To further verify the physiological roles of PPAR*γ* and ANGPTL4 in trophoblast cell invasion and migration, placenta villous explants were performed. As expected, rosiglitazone and rhANGPTL4 markedly promoted the outgrowth of placenta villous explants compared with the controls, and deletion of ANGPTL4 abolished the effects of rosiglitazone (Figures 5f and g). The expression of MMP-2 and MMP-9 was also measured by gelatin zymography, which indicated that their expression, as well as that of TIMP-1 and TIMP-2 was consistent with previous results (Figure 5h). Furthermore, IHC analysis revealed decreased expression of MMP-2 and MMP-9 in preeclamptic placentae (*n*=30) compared with normal placentae (*n*=30) (Figure 5i). These observations collectively demonstrate that ANGPTL4 mediates the PPAR*γ* agonist-induced migration and invasion of trophoblast cells and placental explant outgrowth via regulating MMP-2 and MMP-9.

### ANGPTL4 is involved in PPAR*γ* agonist-induced angiogenesis

**Figure 6:**
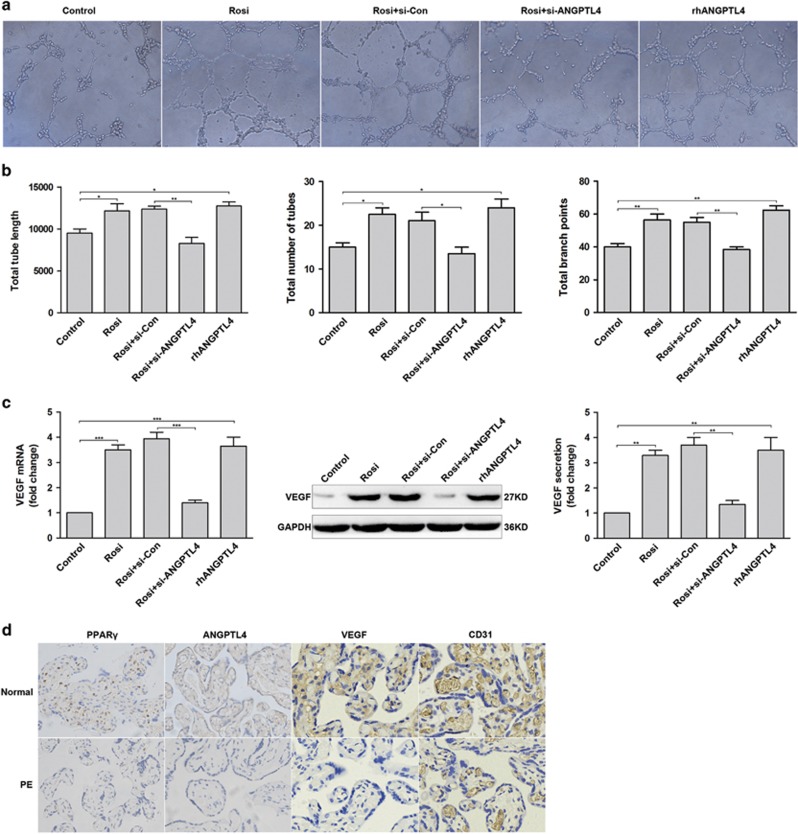
ANGPTL4 plays an important role in rosiglitazone-induced angiogenesis. (**a**–**c**) HUVECs transfected with si-Con or si-ANGPTL4 were stimulated with 1 *μ*M rosiglitazone. Control cells were treated with 1 *μ*M rosiglitazone or 100 nM rhANGPTL4. Tube formation assay was performed in these cells, and then the mRNA and protein levels of VEGF and its secretion were evaluated through western blot, qRT-PCR and ELISA. The data are shown as the means±S.E.M. *P<0.05, **P<0.01 and ***P<0.001 relative to control or si-Con. (**d**) The expression of PPAR*γ*, ANGPTL4, VEGF and CD31 in placental tissues was examined by IHC in PE subjects (*n*=30) and normal controls (*n*=30). Representative images were captured at × 400 magnification

Limited remodelling of the spiral arteries is a main pathological feature of PE.^3^ To explore the roles of PPAR*γ* and ANGPTL4 in angiogenesis, a tube formation assay was performed on HUVECs transfected with si-Con or si-ANGPTL4 and control cells. Rosiglitazone and rhANGPTL4 distinctly increased the total tube length, total number of tubes and total branch points compared with controls, but depletion of ANGPTL4 eliminated the effects of rosiglitazone in angiogenesis (Figures 6a and b). Moreover, the expression and secretion of vascular endothelial growth factor (VEGF) was examined via qRT-PCR, western blot and ELISA, which yielded results similar to those from the tube formation assay (Figure 6c). Eventually, the expression of CD31 (an indicator of vascularity) and VEGF in placental tissues was evaluated by IHC, which indicated their decreased expression in preeclamptic placentae (*n*=30) compared with normal placentae (*n*=30) (Figure 6d). All in all, these data suggest that ANGPTL4 is an important mediator of PPAR*γ* agonist-induced angiogenesis.

## Discussion

**Figure 7:**
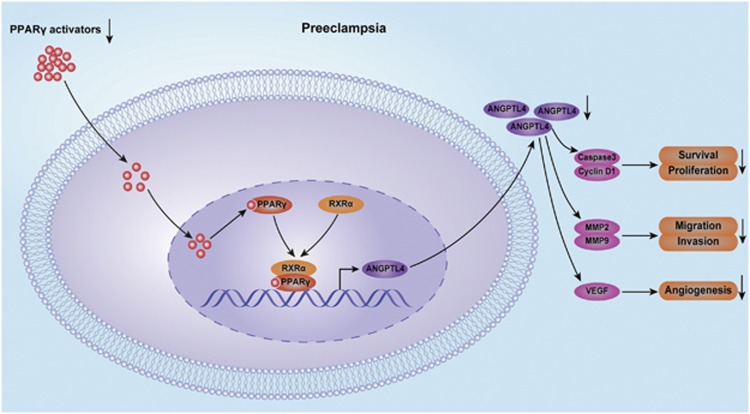
Schematic diagram of ANGPTL4 mediated the protective role of PPAR*γ* activators in the pathogenesis of PE. PPAR*γ* agonist induces ANGPTL4 expression by regulating the binding of the PPAR*γ*/RXR*α* heterodimer to its promoter region and enhancing its transcriptional activity. Moreover, our data show that the expression and circulating activators of PPAR*γ* and the expression and secretion of ANGPTL4 are decreased in PE subjects. In addition, we provide evidence that ANGPTL4 mediates the facilitative effects of the PPAR*γ* agonist on regulating the survival, proliferation, migration and invasion of HTR8/SVneo cells, placental explant outgrowth and angiogenesis in HUVECs

In the present study, we show that PPAR*γ* is a prerequisite for the PPAR*γ* agonist-induced expression and secretion of ANGPTL4 in HTR8/SVneo cells, HUVECs and placental explants in a concentration-dependent manner. We also identify ANGPTL4 as a direct transcriptional target of PPAR*γ* and demonstrate for the first time that decreased expression and secretion of ANGPTL4 may result from a decrease in circulating PPAR*γ* activators in preeclamptic patients compared with normal pregnant subjects. In addition, we provide evidence that ANGPTL4 mediates the facilitative effects of PPAR*γ* agonist-induced survival, proliferation, migration and invasion in trophoblast cells, as well as outgrowth in placental explants and angiogenesis in HUVECs. In conclusion, these findings indicate that ANGPTL4 is involved in the protective effects of PPAR*γ* activators on the pathogenesis of PE (Figure 7).

PPARs are ligand-activated transcriptional factors that regulate many genes associated with cell proliferation and differentiation.^4^ PPAR*γ* is crucial for the differentiation of intricate trophoblast lineages and normal vascular function.^5, 6, 7^ Moreover, previous studies have demonstrated that ANGPTL4 serves as a multifunctional factor involved in the regulation of lipid metabolism, wound healing and angiogenesis.^11, 12^ However, it is not clear whether the expression of ANGPTL4 is regulated by PPAR*γ* agonists in HTR8/SVneo cells, HUVECs and placental explants. Our study demonstrates that the expression and secretion of ANGPTL4 in these cells and placental explants are significantly upregulated in a dose-dependent manner by the PPAR*γ* agonist rosiglitazone. We also found that PPAR*γ* is induced by rosiglitazone in the same manner and is indispensable for the rosiglitazone-induced expression and secretion of ANGPTL4. Our results are similar to those reported previously in which rosiglitazone was determined to increase the expression of PPAR*γ* and ANGPTL4.^18, 19^ It has been reported that ANGPTL4 is a target gene of PPAR*γ*,^20^ and ligand-activated PPAR*γ* regulates the expression of genes via binding to the PPRE of target genes as a heterodimer with RXRs.^15^ Therefore, additional studies were performed to elucidate the molecular mechanisms implicated in ANGPTL4 expression induced by rosiglitazone. We provide evidence in HTR8/SVneo cells, HUVECs and placental explants that RXR*α* is required for the rosiglitazone-induced expression and secretion of ANGPTL4. In addition, results from our ChIP assay also indicate that the PPAR*γ*/RXR*α* heterodimer activates ANGPTL4 transcription by directly binding to PPRE3, located at −233/−209 in its promoter.

PPAR*γ* is required for normal placental development, and its activation regulates trophoblast differentiation and invasion.^7, 21, 22^ Previous studies have shown that ANGPTL4 is involved in the regulation of lipid metabolism, wound healing and angiogenesis.^11, 12^ Nevertheless, the expression of PPAR*γ* and ANGPTL4 and their correlation are not well characterised in PE and normal pregnancies. We demonstrated in placental tissues and serum that the expression of PPAR*γ* and the expression and secretion of ANGPTL4 were significantly reduced in PE compared with normal control subjects. Our findings that PPAR*γ* expression is decreased in PE subjects are supported by those described previously.^2^ In addition, the present study showed that the PPAR*γ* mRNA level is positively correlated with the mRNA level and secretion of ANGPTL4 among normal controls and PE women. A recent study showed that PPAR*γ* activators exist in serum obtained from normal pregnant women and upregulate the expression of PPAR*γ*.^23^ This prompted us to investigate the level of PPAR*γ* activators in the serum among PE and normal control subjects. Placental explants treated with serum from five normal and five PE women exhibited upregulated expression of PPAR*γ* and ANGPTL4 in a concentration-dependent manner. These results indicate that PPAR*γ* expression in placental tissue reflects the serum levels of corresponding PPAR*γ* activators in normal controls and PE subjects. Taken together, our findings suggest that the levels of PPAR*γ* activators in the serum are decreased in women with PE. It is important to note that these results are in good agreement with previous observations.^8^ In addition, decreased PPAR*γ* activators in the serum may be responsible for the decrease in PPAR*γ* and the expression and secretion of ANGPTL4 in PE.

It is widely accepted that PE is associated with excessive apoptosis and poor invasion of trophoblast cells and limited remodelling of spiral arteries.^3^ Therefore, we sought to explore the functional roles of PPAR*γ* and ANGPTL4 in these aspects. Oxidative stress is responsible for increased trophoblast cell apoptosis.^17^ In the present study, hydrogen peroxide, an oxidative stress marker, was used to treat HTR8/SVneo cells. The results showed that ANGPTL4 mediated the role of rosiglitazone in anti-apoptosis, accompanying with the decreased expression of genes associated with apoptosis (cleaved PARP, caspase-9, cleaved caspase-9, caspase-3, cleaved caspase-3 and Bax). Similar to previous findings,^14^ we found that ANGPTL4 mediated the facilitative effects of rosiglitazone on trophoblast cells proliferation, with increased expression of proliferation-related genes (Cyclin D1, Bcl-2, c-Myc and pHH3). Moreover, IHC revealed that preeclamptic placental tissue with low expression of PPAR*γ* and ANGPTL4 displayed increased caspase-3 expression and decreased Cyclin D1 expression compared with normal controls.

Given that poor invasion of trophoblast cells and limited remodelling of the spiral arteries are the main pathological features of PE.^3^ For migration and invasion, we found that ANGPTL4 mediated the PPAR*γ* agonist-induced the migration and invasion of trophoblast cells and placental explant outgrowth. MMPs are required to create a microenvironment that enables the migration and invasion of trophoblast cells, and decreased MMP-2 and MMP-9 expression in preeclamptic placental tissue has been demonstrated.^24, 25^ Analogously, our results suggest that ANGPTL4 mediates PPAR*γ* agonist-induced MMP-2 and MMP-9 expression. Moreover, IHC analysis verified decreased MMP-2 and MMP-9 expression among preeclamptic placental tissues with lower PPAR*γ* and ANGPTL4 levels. VEGF serves as a predominant regulator of angiogenesis and plays a crucial role in the production of blood vessels.^26^ Meanwhile, PE is associated with decreased serum level of VEGF and lower microvessel counts in placentas compared with normal controls.^27, 28^ Previous studies also provide evidence that ANGPTL4 mediates PPAR*γ* agonist-induced angiogenesis.^13, 18^ These data are consistent with our present findings in which ANGPTL4 was determined to be involved in PPAR*γ* agonist-induced angiogenesis by regulating VEGF expression. In addition, decreased expression of VEGF and CD31 were observed in preeclamptic placental tissues with low PPAR*γ* and ANGPTL4 expression.

In summary, we demonstrate that the PPAR*γ* agonist induces ANGPTL4 expression by regulating the binding of the PPAR*γ*/RXR*α* heterodimer to its promoter region and enhancing its transcriptional activity. Moreover, our data show that the expression and circulating activators of PPAR*γ* and the expression and secretion of ANGPTL4 are decreased in PE subjects. It is worth noting that reduced ANGPTL4 expression and secretion may be attributed to decreased circulating activators of PPAR*γ* in PE. In addition, we provide evidence that ANGPTL4 mediates the facilitative effects of the PPAR*γ* agonist on regulating the survival, proliferation, migration and invasion of HTR8/SVneo cells, placental explant outgrowth and angiogenesis in HUVECs. Previous study has reported that rosiglitazone administration ameliorated hypertension, improved vascular function and prevented the development of several of the pathophysiological characteristics associated with the rat model of PE.^9^ Therefore, PPAR*γ* and ANGPTL4 may become potential novel targets for prevention and treatment of PE. However, further studies are required to investigate if PPAR*γ* agonist is effective in prevention and treatment of women with PE.

## Materials and methods

### Collection of placental tissues and blood samples

A total of 60 placental tissues and corresponding blood samples, including 30 PE and 30 normal controls, were collected at Ren Ji Hospital, School of Medicine, Shanghai Jiao Tong University. All placental tissues and blood samples were collected according to protocols approved by the Research Ethic Committee of the School of Medicine, Shanghai Jiao Tong University. Written informed consent was obtained from all patients. The clinical features of patients were displayed in Supplementary Table 1.

### Cell and placental explant culture

HTR8/SVneo (an immortalised human trophoblast cell line) and HUVECs were cultured in DMEM/F12 supplemented with 10% FBS. Placental explants were cultured as previously described.^29, 30^ In brief, tissues from first-trimester placental villi were dissected and explanted onto transwell inserts or a 24-well culture dish pre-coated with Matrigel. Explants were permitted to attach to the Matrigel for 4 h and were then supplied with culture medium without serum. Explants were treated with disparate approaches based on the experimental purpose.

### RNA extraction and qRT-PCR

Total cellular RNA was extracted using TRIzol reagent according to the manufacturer's instructions. Primers for PPAR*γ*, ANGPTL4, VEGF, Cyclin D1, GAPDH and caspase-3 were obtained from Invitrogen Bioengineering Corporation (Shanghai, China). The sequences of the primers being used for PCR reactions were listed in Supplementary Table 2. qRT-PCR was performed as previously described.^31^

### Western blot analysis

Protein preparation and western blot analysis were performed as previously described.^31^ Antibodies against CK7, PPAR*γ*, ANGPTL4, RXR*α*, Cyclin D1, Bax, Bcl-2, caspase-3, caspase-9, cleaved caspase-3, cleaved PARP, c-Myc, pHH3, MMP-2, MMP-9, TIMP-1, TIMP-2, *β*-actin and GAPDH were obtained from Abcam (Cambridge, UK). Antibodies against VEGF and cleaved caspase-9 were obtained from Millipore (Billerica, MA, USA) and Cell Signalling Technology (Boston, MA, USA), respectively.

### ELISA

ELISAs (Abcam) were used to detect the levels of ANGPTL4 and VEGF and were performed according to the manufacturer’s instructions. The concentration of each sample was determined by measuring the absorbance at 450 nm in a microplate reader. Each sample was run in triplicate.

### Immunohistochemical and immunofluorescence staining

IHC and immunofluorescence were performed as previously described.^32^ For immunofluorescence analysis, whole mount immunofluorescent staining was performed to confirm the role of PPAR*γ* in ANGPTL4 expression in placental explants. In brief, placental explants cultured for 72 h in the presence of phenol red-free Matrigel were fixed with 4% paraformaldehyde. Subsequently, the explants were washed, blocked, and incubated with the primary antibody and fluorescent secondary antibodies. Ultimately, the fluorescent signals were photographed on an inverted microscope.

### ChIP

ChIP was carried out as previously described.^33^ Three sets of PCR primers were designed to represent different regions of the ANGPTL4 promoter: PPRE1, PPRE2 and PPRE3. Primer sequences were shown in Supplementary Table 2.

### TUNEL and cell proliferation assays

The TUNEL assay was performed according to the manufacturer’s instructions (Sigma-Aldrich, St. Louis, MO, USA). Cell proliferation was determined using the Cell Counting Kit-8 (CCK-8) assay (Dojindo, Rockville, MD, USA) according to the manufacturer’s instructions.

### Transwell invasion and wound-healing assays

Wound healing and transwell invasion assays were executed as previously described.^34^ Cell invasion was detected using a cell culture insert (Corning Incorporated, USA) coated with Matrigel (BD Biosciences, San Jose, CA, USA).

### Gelatin zymography assay

Gelatin zymography was carried out as previously reported.^32^ In brief, the supernatants were collected and electrophoresed on SDS-polyacrylamide gels with 1% gelatin. Subsequently, the gels were washed, incubated, stained and destained successively.

### Tube formation assay

The tube formation assay was performed as follows: growth factor reduced Matrigel (BD Biosciences) was placed in a 96-well cell culture plate (60 *μ*l/well) and incubated at 37 °C for 30 min. HUVECs transfected with si-Con or si-ANGPTL4 (10 000/200 *μ*l) were seeded onto the Matrigel-coated wells. HUVECs served as a control. Cells were treated with rosiglitazone or recombinant human ANGPTL4 and incubated at 37 °C for 20 h. Tube formation was observed under an inverted microscope.

### Statistical analysis

All data are shown as mean±S.E.M. All statistical analyses, including one-way ANOVA, *t*-test and Pearson’s correlation, were executed using SPSS 20.0. All experiments were independently repeated at least in triplicate. Differences between groups were thought to be statistically significant at *P*<0.05.
