# Supplementary figures and images for: ANGPTL4 mediates the protective role of PPARγ activators in the pathogenesis of preeclampsia
Source: Cell Death Dis. 2017 Sep 21;8(9):e3054–. doi: 10.1038/cddis.2017.419 (PMC5636970; doi:10.1038/cddis.2017.419)

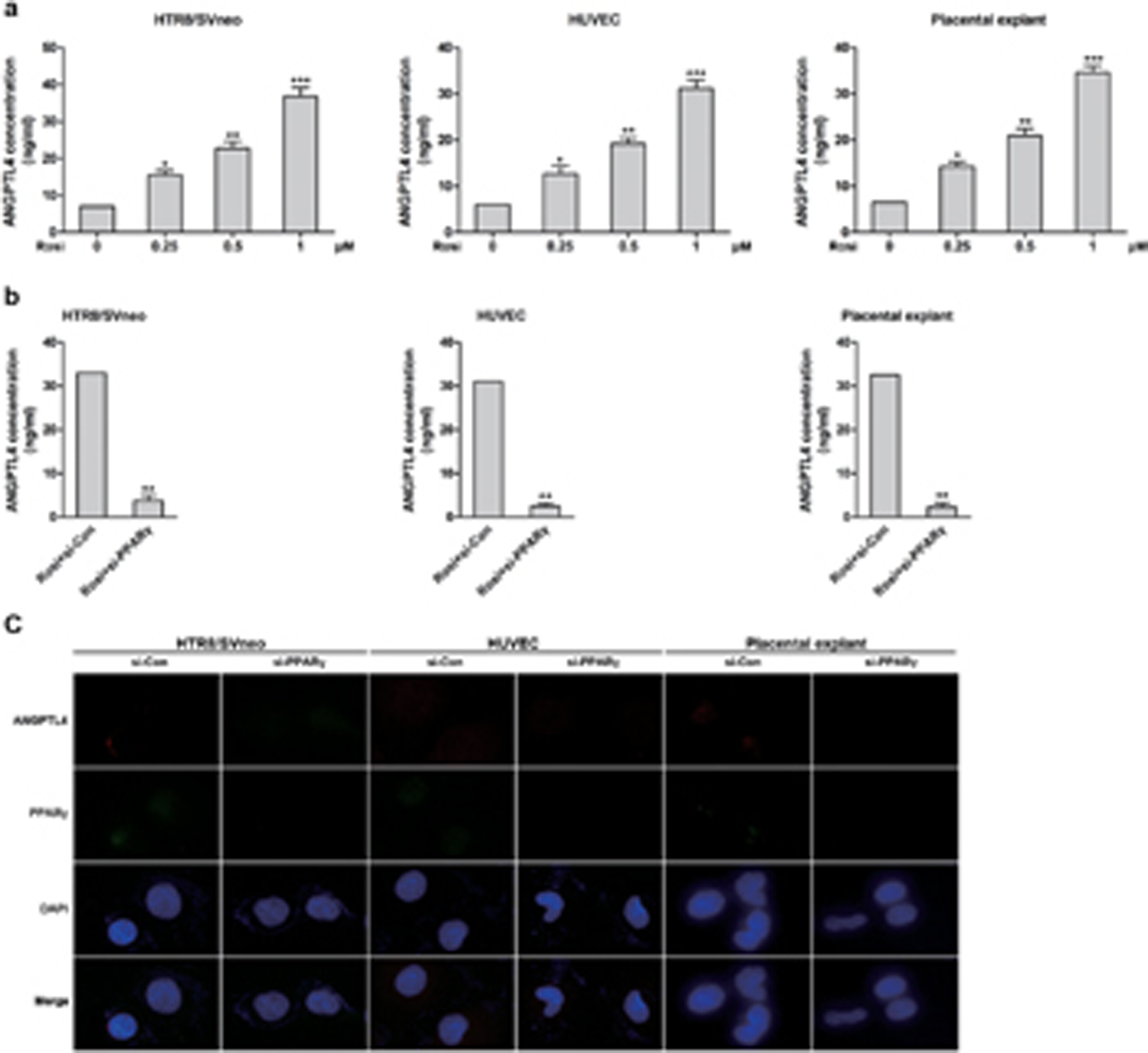

Supplement: Supplementary Figure 1 [file cddis2017419x1.tif]

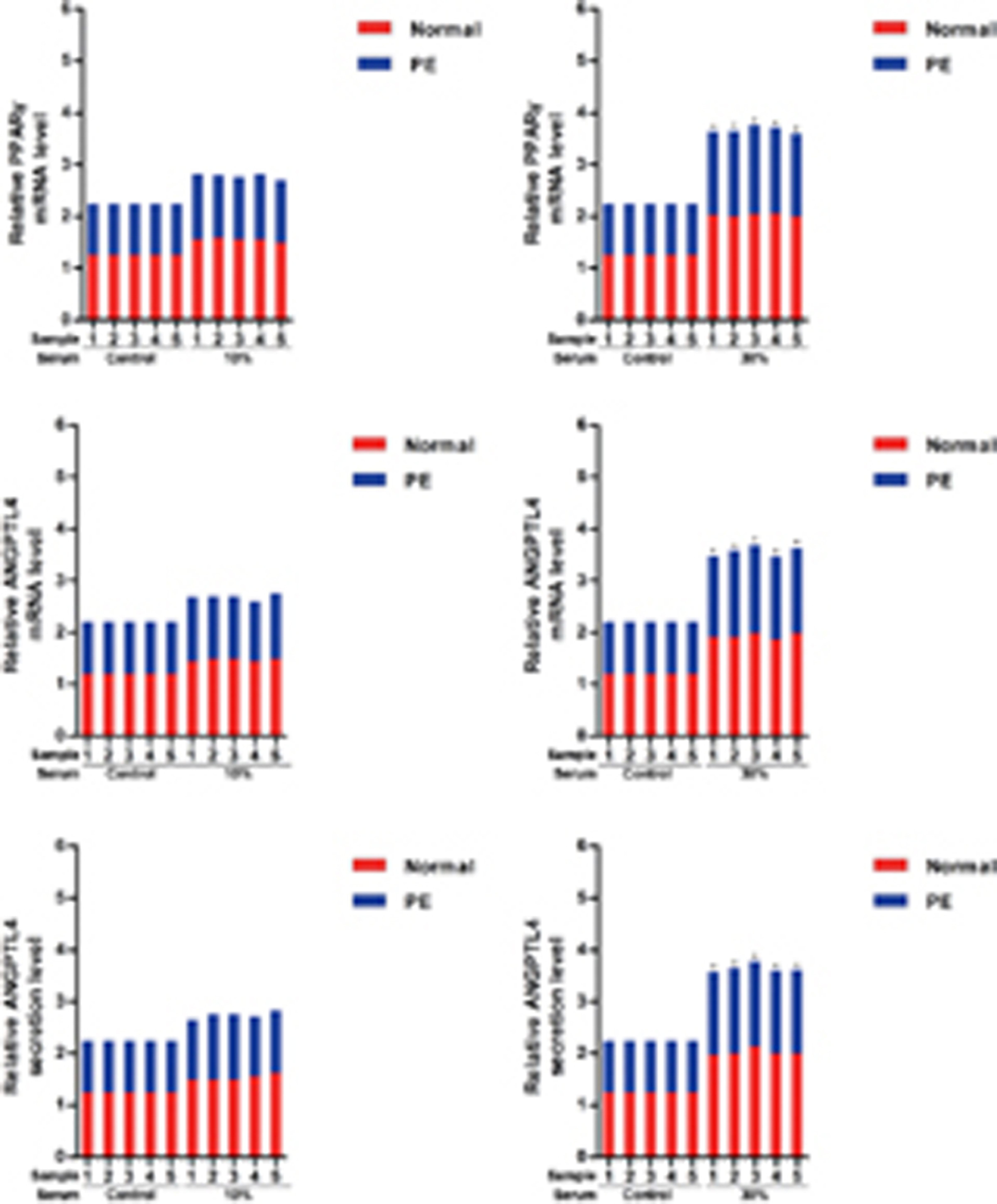

Supplement: Supplementary Figure 2 [file cddis2017419x2.tif]

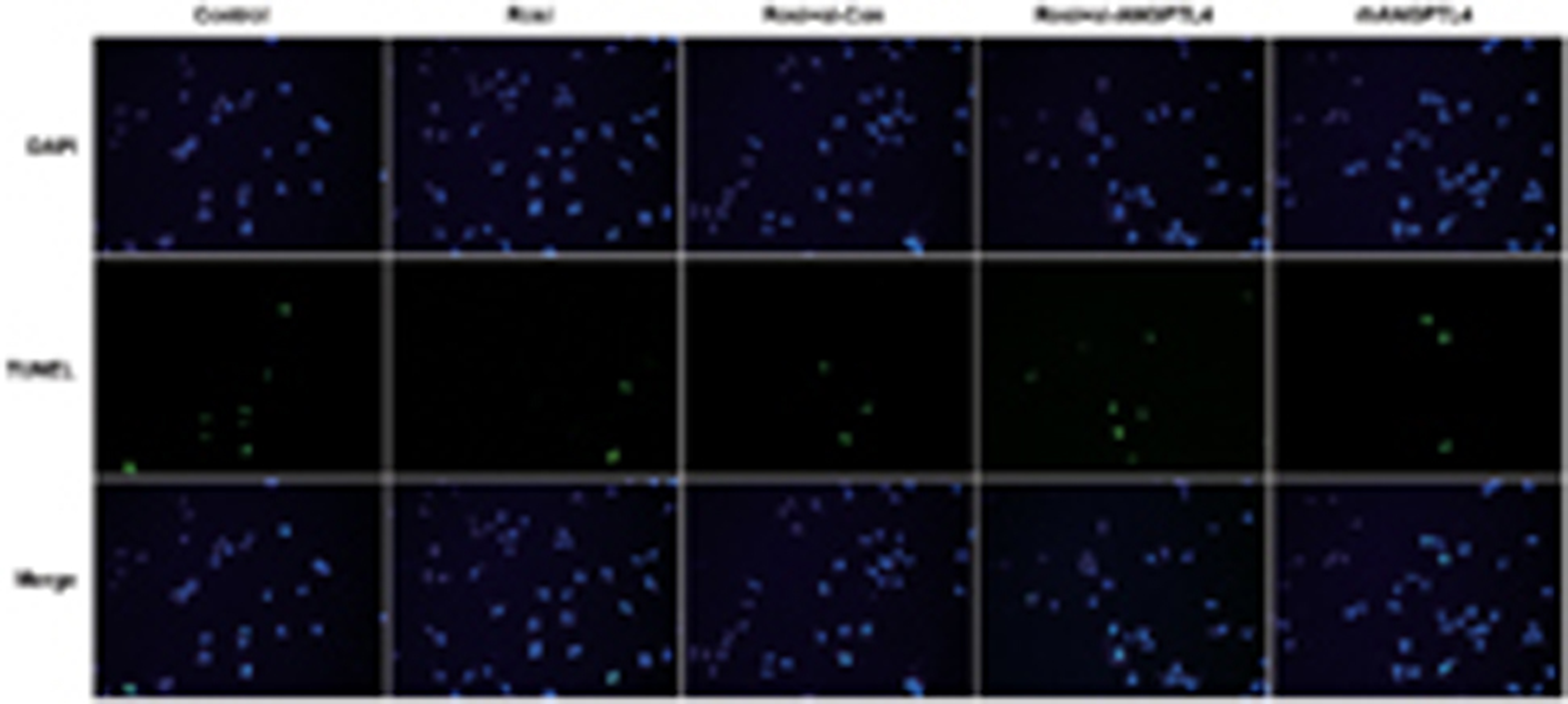

Supplement: Supplementary Figure 3 [file cddis2017419x3.tif]
