# Supplementary material for: ANGPTL4 mediates the protective role of PPARγ activators in the pathogenesis of preeclampsia
Source: Cell Death Dis. 2017 Sep 21;8(9):e3054–. doi: 10.1038/cddis.2017.419 (PMC5636970; doi:10.1038/cddis.2017.419)
Supplement: Supplementary Figure Legends [file cddis2017419x4.docx]

**Supplementary Figure legends**

**Supplementary Figure 1** Rosiglitazone stimulates the expression and secretion of ANGPTL4 via PPARγ. (**a**) HTR8/SVneo cells, HUVECs and placental explants were stimulated with Rosi (rosiglitazone) (0, 0.25, 0.5, and 1 μM) for 18 hours. The secretion of ANGPTL4 was determined by ELISA. The data are shown as the means ± S.E.M. **P*<0.05, ***P*<0.01 and ****P*<0.001 compared to control. (**b**) HTR8/SVneo cells, HUVECs and placental explants were transfected with control siRNA (si-Con) or PPARγ siRNA (si-PPARγ), and then treated with 1 μM rosiglitazone for 18 hours. The secretion of ANGPTL4 was evaluated by ELISA. The data are shown as the means ± S.E.M. ***P*<0.01 relative to corresponding control (**c**) HTR8/SVneo cells, HUVECs and placental explants were transfected with si-Con or si-PPARγ, and then cultured for 18 hours. The expression of PPARγ and ANGPTL4 was evaluated by immunofluorescence. Representative images are shown.

**Supplementary Figure 2** The combined data from Figure 3j and k was analyzed. The data are shown as the means. **P*<0.05 compared to PE.

**Supplementary Figure 3** HTR8/SVneo cells transfected with control siRNA (si-Con) or ANGPTL4 siRNA (si-ANGPTL4) were stimulated with 1 μM rosiglitazone in the presence of 150 μM hydrogen peroxide. Control cells were treated with 1 μM rosiglitazone or 100 nM rhANGPTL4 in the presence of 150 μM hydrogen peroxide. A TUNEL assay was performed to evaluate the rate of cellular apoptosis. The apoptotic rate was expressed as the ratio of the number of the cells with TUNEL-positive nuclei (green) to the total number of DAPI-positive (blue) cells. Representative images are shown.
