# Supplementary material for: ANGPTL4 mediates the protective role of PPARγ activators in the pathogenesis of preeclampsia
Source: Cell Death Dis. 2017 Sep 21;8(9):e3054–. doi: 10.1038/cddis.2017.419 (PMC5636970; doi:10.1038/cddis.2017.419)
Supplement: Supplementary Table [file cddis2017419x5.docx]

**Supplementary Table**

**Supplementary Table 1.** Clinical characteristics of the pregnant women enrolled in this study

|  | Normal pregnancy (n = 30) | Preeclampsia (n = 30) | *P* value |
| --- | --- | --- | --- |
| Maternal age, y  BMI, kg/m^2^  Gestational age, wk  BP, mm Hg  Systolic  Diastolic  Proteinuria, g/24h  Infant birth weight , g | 28.7 ± 2.2  27.7 ± 1.2  38.9 ± 0.8  122.0 ± 3.1  77.0 ± 3.8  NA  3442 ± 213 | 30.7 ± 3.5  30.0 ± 2.5  37.4 ± 0.3  155.7 ± 4.1^*^  105.3 ± 4.4^*^  2.8 ± 0.6  3074 ± 328 | 0.65  0.45  0.16  <0.01  <0.01  NA  0.40 |

Data are shown in mean ± SEM.

**P*<0.01 compared with normal pregnancy.

Abbreviation: BMI, body mass index; BP, blood pressure; NA, not available.

**Supplementary Table 2.** List of primers used in this study

| Gene Name | Sequence (5' to 3') |
| --- | --- |
| PPARγ  ANGPTL4  Caspase3  Cyclin D1  VEGF  GAPDH  PPRE1  PPRE2  PPRE3 | F: CCTCATGGCAATTGAATGTCG  R: CCGGAAGAAACCCTTGCATC  F: GGACCACAAGCACCTAGACCA  R: GATCCCCAAACCCCGCCTT  F: CCCATTTCTCCATACGCACT  R: TTCACTTTCTTACTTGGCGAT  F: CCCTCCAGAACACGGCTCA  R: CTTGCCCCATCACGACAGA  F: AGATTATGCGGATCAAACCTC  R: GATTTCTTGCGCTTTCGTT  F: CCATCACCATCTTCCAGGAG  R: CCTGCTTCACCACCTTCTTG  F: GTGTGCAGTTTCAGATTCCA  R: TCATTCAAACTTGCACCGAT  F: CGAGCCCAGAAAAGTCTCT  R: CAGTAGAATCGCTTGAACCC  F: CATCTCGGCGCACTGCAAC  R: CGCCTCTAGTGTGAAATTGCAAG |
